# What are NLRP3-ASC specks? an experimental progress of 22 years of inflammasome research

**DOI:** 10.3389/fimmu.2023.1188864

**Published:** 2023-07-26

**Authors:** Abhinit Nagar, Ravi Bharadwaj, Mohammad Omar Faruk Shaikh, Abhishek Roy

**Affiliations:** ^1^Department of Flow Cytometry, Cytek Biosciences, Fremont, CA, United States; ^2^MassBiologics of the University of Massachusetts Medical School, Boston, MA, United States; ^3^Division of Critical Care Medicine, Department of Anesthesiology, Critical Care and Pain Medicine, Boston Children’s Hospital, Boston, MA, United States; ^4^Department of Anesthesia, Harvard Medical School, Boston, MA, United States; ^5^Department of Physiology and Biomedical Engineering, Mayo Clinic, Rochester, MN, United States

**Keywords:** ASC specks, SMOCs, Casp-1, NLRP3, inflammasome

## Abstract

Speck assembly is the hallmark of NLRP3 inflammasome activation. The 1µm structure comprising of NLRP3 and ASC is the first observable phenotype of NLRP3 activation. While the common consensus is that the specks are the site of inflammasome activity, no direct experimental evidence exists to support this notion. In these 22 years, since the inflammasome discovery, several research studies have been published which directly or indirectly support or refute the idea of speck being the inflammasome. This review compiles the data from two decades of research to answer a long-standing question: “What are NLRP3-ASC specks?”

## Introduction

Nod-like receptor protein containing pyrin 3 (NLRP3) is a cytosolic pathogen recognition receptor (PRR) predominantly expressed in myeloid cells (1, 2). It detects a wide range of chemically and structurally diverse stimuli and initiates an inflammatory response by forming multi-protein complexes called inflammasomes (1, 3, 4). Upon stimulation, NLRP3 recruits an adaptor protein called apoptosis-associated speck-like protein containing a caspase recruitment domain (ASC) and pro-caspase-1. Caspase-1 activation leads to the processing of IL-1β, IL-18, and Gasdermin-D (GSDMD), which contribute to the inflammatory response against various threats (1, 3, 4). The inflammasome complexes, consisting of NLRs, ASC, and pro-caspase-1, are present in the high-molecular-weight (HMW) fraction of approximately 700 kDa (5–7). This suggests that the inflammasome may consist of 5-7 NLRP3-ASC-Casp1 units, similar to the apoptosome structure (8–10). Recent research by Xiao et al. revealed that NLRP3 forms a disk-like structure with 10 units, measuring around 32 nm (11). This configuration facilitates caspase-1 activation through proximity-induced interactions (10, 12–14).

Under normal conditions, NLRP3 and ASC are dispersed throughout the cytoplasm (15–17). However, upon NLRP3 activation, they rapidly relocate to form a perinuclear punctate structure known as the “speck” (15, 18). The speck, approximately 1 μm in diameter, can be visualized using fluorescence microscopy (9, 16, 17, 19). Speck formation concentrates the majority of inflammasome components and is crucial for the inflammasome response (20–23). Disruption of NLRP3:ASC interactions by mutations or small-molecule drugs impairs speck formation and inflammasome function (24–28). Colchicine, a drug that inhibits speck formation, is commonly used to treat inflammasome-related conditions like gout (4).

Although the speck is considered the site of inflammasome activity, its size is significantly larger than the observed size of an inflammasome (10, 17, 26). Additionally, some studies have shown caspase-1 activation in the absence of speck formation, challenging the notion that specks are inflammasomes (9, 17, 25, 29). Nonetheless, due to its rapid formation, recruitment of free NLRP3 and ASC, and its nature as a multi-protein complex, the speck is generally believed to represent the inflammasome.

This review aims to explore the research conducted in the field of inflammasomes, focusing on the nature and relevance of inflammasome-associated specks in the physiological functioning of the inflammasome. Recent studies have suggested that ASC specks exhibit prion-like polymerization and Supramolecular Organizing Center (SMOC)-like threshold properties, which enhance optimal inflammasome activation even with weaker stimuli. However, despite 22 years of inflammasome research, the direct experimental evidence supporting the speck as the inflammasome remains elusive, making it one of the biggest mysteries in inflammasome biology.

## Inflammasome complex assembly

The relationship between speck assembly and NLRP3 activation remains complex, with multiple proposed models adding to the confusion (10, 14, 17, 20, 25–27, 30, 31). The precise mechanism by which NLRP3 and other inflammasome components assemble to form a functional multi-protein complex is still unclear. A comprehensive review by Elliot et al. has discussed the limitations of these models (8). Broadly, the proposed ideas can be categorized into two distinct models: the complex assembly model and the pre-assembled complex activation model.

**Figure 1:**
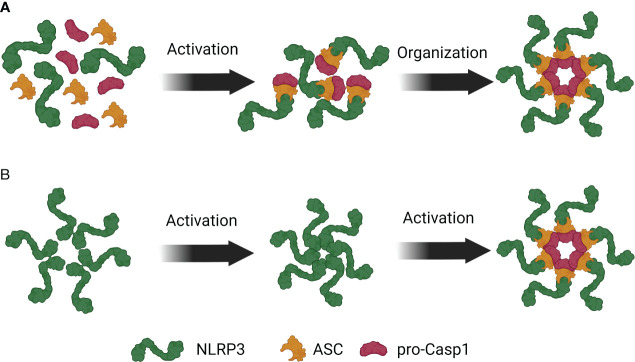
Competing models of NLRP3 inflammasome assembly. **(A)** Upon NLRP3 activation, ASC binds to NLRP3 and facilitates recruitment of pro-Caspase-1 to the complex. These trimeric complexes then oligomerize to form an inflammasome. **(B)** NLRP3 is present in the cytosol as a pre-formed inactive oligomeric complex where LRRs of NLRP3 are near the PYD. Activation induced structural changes in NLRP3 facilitate the recruitment of ASC and pro-Caspase-1 to the complex.

The complex assembly model suggests that upon activation, NLRP3 recruits caspase-1 via ASC to form monomeric inflammasome units. These units then oligomerize through the Nucleotide Binding Domain (NBD) to create a functional inflammasome complex (**Figure 1A**). This model is supported by the observation that nearly all NLRP3 and ASC associate to form a singular speck. Furthermore, active NLRP3 co-immunoprecipitates with ASC and pro-caspase-1 (14, 27) and elutes with other components in complexes larger than 700 kDa (5, 6). *In vitro* studies have identified complexes smaller than a speck that contain caspase-1 (5, 6). However, whether these monomeric units further oligomerize to form a speck remains uncertain. Notably, these complexes exhibit different stoichiometry of monomeric units (11, 32), with mouse NLRP3 inflammasomes displaying a double-caged model of six units and human NLRP3 inflammasomes forming a disk shape composed of ten units (11). These species-specific differences raise questions about the unified activation of NLRP3 inflammasomes across different organisms and the function of complexes with varying monomeric units.

The counter-model proposes that a resting pre-assembled NLRP3 complex undergoes a conformational change upon activation and recruits ASC and pro-caspase-1 to the complex (**Figure 1B**). Under resting condition, NLRP1 and NLRP3 complexes elute in high-molecular-weight (HMW) complexes, indicating their presence in non-stimulated cells (5, 6). Bioluminescence Resonance Energy Transfer (BRET) experiments have shown that the leucine-rich repeats (LRRs) of NLRP3 are closely associated with the PYD domain, suggesting an interaction between them (33). This interaction maintains NLRP3 in an auto-repressed state that is disrupted upon ASC co-expression, indicating a basal level of NLRP3:ASC interaction (33). Furthermore, ASC PYD self-oligomerizes and can participate in pre-activation self-assembly (10, 18, 21, 26, 34).

The contrasting models highlight the complexity of the relationship between inflammasome activation and assembly. Importantly, these observations are made at different stages of inflammasome activation and in various cell types. Therefore, it is likely that both models hold some truth, and multiple states of inflammasome assembly and NLRP3 activation can coexist simultaneously.

## Speck assembly

### Speck assembly: structural dynamics and kinetics

Specks are perinuclear structures formed upon NLRP3 activation (5, 15, 35) and have recently been classified as supramolecular signaling complexes (SMOCs) (31, 36). SMOCs are higher order complexes that promote cooperative assembly of signaling components through weak allosteric interactions, enabling high local concentrations of these components (31, 36–38). SMOC assembly is tightly regulated and plays a role in threshold behavior, temporal and spatial control of signal transduction, and binary all-or-none responses (31, 36–38). However, the specific regulatory mechanisms involved in SMOC-mediated inflammasome activation remain poorly understood. The assembly of SMOCs involves prion-like polymerization, which lowers the Gibb’s free energy (ΔG) of a protein (21, 23, 26, 30), potentially altering protein conformation and kinetics (31, 36–38). Speck formation follows the SMOC assembly mechanism, indicating that specks enable inflammasome proteins to adopt a conformation that efficiently senses the activation signal (20, 22, 26, 39). This efficient signaling may contribute to the threshold and all-or-none nature of inflammasome responses.

While NLRP3 and ASC are unequivocally present within the speck, the presence of other components is still a subject of debate. In THP1 cells, NLRP3 and ASC oligomerizes under three minutes to form a speck making it an incredibly rapid process (35). Similarly, in HeLa cells, the concentration of cytosolic ASC drops from 20μM to around 0.1μM within 100 seconds (34). Similar observations have been made in other cell types like BMDMs and HEK293T cells (40, 41). Interestingly, the kinetics of speck formation differ *in vivo*. In Zebrafish, speck size stabilizes after a continuous growth period of 15 minutes (42). The advantages of such rapid protein oligomerization are not yet clear. Additionally, the morphology of the speck is a topic of discussion. Early studies suggested a hollow to fibrillar structure composed of small protein complexes (15, 18, 43), but recent studies using high-resolution microscopy and simulation models propose that the speck assembly consists of intertwined ASC filaments (37, 38).

These discrepancies may arise from various factors, including experimental conditions, cell types, and organisms. However, the differences highlight our current limitations in understanding speck formation, the regulation of speck assembly, the presence of a single speck per cell, and the mechanisms governing these processes. Some studies have reported the formation of multiple specks per cell (16, 44, 45), suggesting the existence of conditions and mechanisms that promote the formation of more than one speck. Investigating these conditions and mechanisms would be intriguing for further research.

### Speck formation: the molecular view

Speck assembly is characterized traditionally as a microtubule-driven process (31, 36–38, 44, 46, 47). During infection and injury, PAMPs and DAMPs induce damage to the mitochondria which binds to ASC. Damaged-mitochondria laden ASC is transported towards the endoplasmic reticulum (ER) through microtubules, where NLRP3 is located (44). NLRP3 and ASC being in proximity interacts and colocalize to form one cytosolic perinuclear speck of 1 µm (35, 48). Microtubule depolymerization agents like colchicine and nocodazole block speck assembly further supporting the idea that microtubules facilitate speck assembly (35, 48). Consistent with speck’s proximity to centrosome, movement and activation of NLRP3 is dependent upon binding with two centrosomal proteins, Microtubule-affinity regulating kinase 4 (MARK4) (47) and NEK7, respectively (49–53). However, some studies suggest that NLRP3 is not localized exclusively on the centrosome (54) but also interacts with mitochondria (44, 55–62), Golgi apparatus (63, 64) and redox-associated proteins like TXNIP (62, 65). Thus, whether NLRP3 interaction with the centrosome is essential is still unclear. These differences can be the result of using different cell types and stimulation of NLRP3 by different agonists. It is likely that NLRP3 shuttles between different organelles or a sub-population of NLRP3 binds to one organelle while another sub-population binds to other organelle. However, it is still not clear what signals would determine shuttling from or the preference to bind one organelle to another. Recently few papers have shown the role of membrane association of NLRP3 in inflammasome formation. Membrane-bound NLRP3 promotes ASC oligomerization and inflammasome activation (63). Moreover, NLRP3 forms a double-ring cage structure facilitated by LRR interaction. Such LRR interactions are also required to disperse Trans-Golgi network (TGNs) into vesicles (63). In these studies, they have also revealed that a fraction of membrane unbound NLRP3 is also present in the cells which constitute the inactive form of NLRP3.

## Specks as the site of inflammasome activity

NLRP3 activation results in rapid relocation of NLRP3 and ASC to form a speck, which is followed by caspase-1 activation suggesting that speck formation is required for inflammasome formation (1, 9, 16, 17, 20, 66). The idea that specks are needed for the inflammasomes stems from several observations. Firstly, speck formation is the first event which happens within 3 minutes of NLRP3 activation (19, 39, 40, 67, 68) and thus precedes inflammasome formation. Since caspase-1 is activated through proximity induced dimerization and only one speck is formed per cell, it is likely that caspase-1 activation occurs inside the speck (10, 12, 14). Secondly, small molecule drug, like MCC950, that inhibit specks also impair the NLRP3 inflammasome response (24, 28) suggesting that speck formation is necessary for inflammasome activation. MCC950 inhibit speck formation, specifically block NLRP3 inflammasomes in human and mouse macrophages and alleviate symptoms of Cryopyrin Associate Periodic Syndrome (CAPS) in a mouse model. Further, colchicine is used to treat gout (69) and apart from blocking speck formation (44), it also impairs IL-1β maturation after MSU stimulation (70). This finding suggests that speck assembly is required for inflammasome formation and/or possibly can be the site of inflammasome function, i.e., caspase-1 activation and subsequent IL-1β maturation. Thirdly, mutations that disrupt speck assembly also impair inflammasome function (25–27) further implicating that speck assembly is required for inflammasome function. Although indirect, but these observations strongly suggest that speck is the inflammasome. Conversely, these observations also suggest that preventing specks blocks the downstream events leading to inflammasome formation. Lastly, few studies have noted that active caspase-1 colocalizes with NLRP3 and ASC at the speck structure (2, 71) providing direct evidence of presence of caspase-1 at the speck. Several other tangential evidence exists that can be extrapolated to say that speck is the inflammasome, but none of such evidence directly establishes any such relationship.

## Disparity between speck structure and inflammasomes

Contradictory to the idea that specks are inflammasomes, some observations suggests that specks may have some different roles. The reported size and stoichiometry of specks differ significantly from known inflammasome structures (10, 11, 20, 23, 26, 35, 39, 72, 73). Specks are approximately 100-1000-fold larger and display variations in the ratio of NLR, ASC, and Caspase-1 compared to other inflammasome complexes (10, 15, 71, 74). Discrepancies in stoichiometry (10, 26) can be attributed to the different proteins used in the studies, where full-sized NLRPs and ASC were used by Faustin et al. (10), while Lu et al. and Sborgi et al. used isolated PYD and CARD domains of ASC (26, 74). The absence of the CARD domain leads to uninterrupted filamentous PYD structures, as observed in these studies (26, 74). These differences in size and composition suggest that specks and inflammasomes are structurally distinct.

Interestingly, caspase-1 activity has also been observed in smaller death complexes that do not resemble specks (17, 25, 75). Moreover, Martinon et al., in their initial paper describing inflammasomes, passed cell lysates through a 0.45 µm filter, excluding the 1 µm specks (6). However, the filtered lysates still activated caspase-1 (6), suggesting that structures smaller than 0.45 µm can function as inflammasomes. Furthermore, specks are 500-1000-fold larger than the cryoelectron micrograph of inflammasomes (10, 26). Calculations based on size and mass relationships suggest that specks, assumed to be toroidal structures, would be approximately 150-15000 times larger than an inflammasome (76). These assumptions may vary depending on the torus’s major-to-minor radius ratio and the assumption of a perfect toroidal structure. While smaller structures resembling inflammasomes have been observed inside specks (26), their similarity to cryoelectron micrograph structures is yet to be established. Further investigations utilizing high-resolution microscopy and advanced biophysical techniques are needed to confirm the presence of these smaller structures in cells.

It is possible that specks are conglomerate structures composed of smaller inflammasomes, which are the sites of caspase-1 activity. Upon NLRP3 activation, these smaller structures may leave the speck, resulting in reduced speck size (2, 17). Interestingly, the gross-morphology of the specks is unaffected by the presence of caspase-1 suggesting that caspase-1 is either not an integral part of the speck or its recruitment is temporally regulated further complicating the relationship between these two structures (2, 17). The presence of caspase-1 within specks remains inconclusive, with some studies demonstrating caspase-1 colocalization while others suggesting only a fraction of active caspase-1 is localized within the speck (2, 71). Even in studies using super-resolution microscopy (71), point-spread function analysis suggests that the ring of the speck which is mostly ASC is in focus, whereas the center which is comprised of active caspase-1 is spread-out above and below the plane suggesting that only a fraction of active caspase-1 may be colocalized. Additionally, speck formation alone is not sufficient for inflammasome function, as specks lacking NLRP3 fail to activate caspase-1 or promote IL-1β maturation (9, 29). Studies have shown that higher doses of stimuli, even in the presence of colchicine, can still activate the inflammasome without inducing speck formation (17, 77). These phenotypes have been observed in *in-vitro* conditions and its physiological relevance are yet to be investigated. It is not clear how such high stimuli conditions manifest in a more relevant physiological condition. It is also possible that higher conditions doses alter the expression levels of inflammasome components which can account for different threshold behavior.

Abovementioned observations, in addition to the presence of multiple caspase-1 activating complexes within cells (17, 25, 75), highlight the complexity of inflammasome activation. Mutations disrupting speck formation without impairing IL-1β production (25), multiple smaller complexes instead of a single speck (44), and multiple caspase-1 activation sites (17) beyond the speck have been observed. Active caspase-1 was found to have low colocalization with specks (16, 17, 45), and live imaging showed caspase-1 activation throughout the cytoplasm rather than in a speck-like location (78). These findings suggest the presence of smaller death-complexes and non-speck locations for caspase-1 activation, challenging the notion that specks are the exclusive site of inflammasome activity.

In summary, conflicting evidence challenges the idea that specks are inflammasomes. The structural and functional distinctions between specks and inflammasomes, the presence of smaller inflammasome-like structures within specks, and the observation of caspase-1 activity outside the speck all contribute to the complexity and ambiguity surrounding the relationship between specks and inflammasome activation. However, it is crucial to conduct these studies under more physiological conditions to minimize potential variations resulting from *in vitro* stimulation. Further research using advanced techniques and physiological settings is required to unravel these intricate mechanisms.

## Discussion

**Figure 2:**
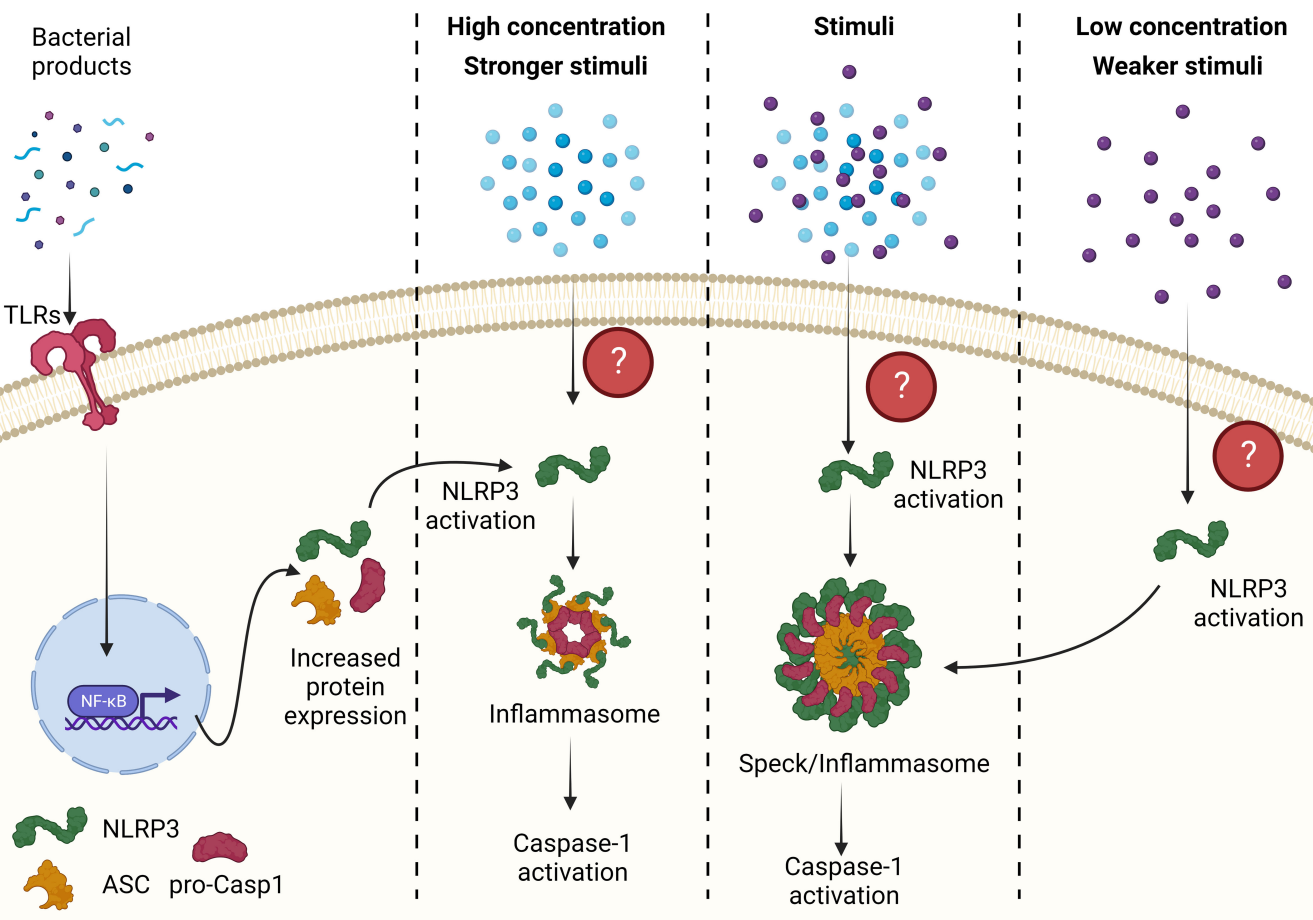
The relationship between an ASC speck and inflammasome. A weaker stimulus or a lower concentration of stimuli induces speck formation, which lower the threshold of NLRP3 activation possibly through prion-like folding of NLRP3, ASC and pro-caspase-1 and formation of smaller inflammasomes. These smaller inflammasome complex are the major activation site of caspase-1. However, in the presence of stronger stimulus or higher concentration of the stimuli, NLRP3 activation does not require prion-like folding and can assemble with ASC and caspase-1 to form inflammasomes. Conversely, if specks are the sole site of caspase-1 activation then any stimuli leading to NLRP3 activation forms a speck where caspase-1 is activated. *All figures are drawn using* BioRender.

The role of specks in inflammasome function remains a subject of debate and requires further investigation. While some researchers propose that specks are the site of caspase-1 activation and inflammasome activity, others suggest that specks may serve as a critical node in the inflammasome activation pathway (Reviewed in Elliot et. al.) (8) (**Figure 2**). To answer the question of what NLRP3-ASC specks truly are, additional experiments utilizing advanced technologies such as imaging flow cytometry (16, 17, 79–81), cryo-electron microscopy, super-resolution microscopy (16, 17, 79–81), BRET and proximity ligation assays (33, 44) are needed.

Studying specks is essential despite caspase-1 activation occurring outside the speck and the presence of smaller death complexes. Investigating specks helps unravel their molecular composition, organization, and role in caspase-1 activation and regulation, shedding light on inflammasome signaling dynamics. Simultaneously, understanding alternative activation sites such as smaller death complexes provide insights into their composition, formation mechanisms, and regulatory roles. Examining the spatial and temporal regulation of caspase-1 activity under different cellular conditions enhances our comprehension of inflammasome signaling complexity. Re-evaluating existing literature in light of current knowledge can identify gaps in inflammasome assembly and ASC speck understanding. In addition to investigating the inflammasome-associated role of ASC specks, it is equally important to explore potential non-inflammasome functions of these structures. To truly comprehend NLRP3-ASC specks, advanced techniques are necessary to evaluate colocalization, modulate activation thresholds, and investigate non-inflammasome functions. These efforts will advance our understanding of specks and their significance in inflammasome biology.

## Author contributions

AN conceptualized and wrote the manuscript. AN, RB, MS and AR edited the manuscript. All authors contributed to the article and approved the submitted version.
